# Protective effect of dehydroandrographolide on obstructive cholestasis in bile duct-ligated mice

**DOI:** 10.18632/oncotarget.21233

**Published:** 2017-09-23

**Authors:** Zhiyong Weng, Xuefeng Liu, Jiehua Hu, Jingzhou Mu, Jing Xie, Chenjuan Yao, Lihua Li

**Affiliations:** ^1^ Department of Cell Biology, Jinzhou Medical University, Jinzhou, PR China; ^2^ Naval University of Engineering, Logistics College, Information Center, Tianjin, PR China; ^3^ Department of Physiology, Dalian Medical University, Dalian, PR China; ^4^ Department of Molecular Oral Physiology, The University of Tokushima Graduate School, Tokushima, Japan

**Keywords:** dehydroandrographolide, obstructive cholestasis, liver adaptive response, anti-fibrosis formation

## Abstract

**Background:**

Dehydroandrographolide (DA) is the main contributor to the therapeutic properties of the medicinal plant Andrographis paniculata (AP). However, it is unknown whether DA has a hepatoprotective effect on obstructive cholestasis in mice and humans.

**Methods:**

We administered DA to mice for 5 days prior to bile duct ligation (BDL) and for the 7 days. Liver function markers, liver histology and necrosis, compensatory responses of hepatocytes, liver fibrosis and the expression of hepatic fibrogenesis markers were evaluated in BDL mice and/or human LX-2 cells.

**Results:**

Mice treated with DA demonstrated lower levels of serum alanine transarninase (ALT), milder liver damage, liver necrosis and fibrosis formation than in vehicle control with carboxymethylcellulose (CMC) mice after BDL. DA treatment also enhanced the Mrp3 expression of hepatocytes but not Mrp4 following BDL. Further, DA treatment in BDL mice significantly reduced liver mRNA and/or protein expression of Tgf-β, Col1a1, α-Sma and Mmp2. This result was also supported by hydroxyproline analysis. The molecular mechanisms of DA treatment were also assessed in human hepatic stellate cell line (LX-2 cell). DA treatment significantly inhibited Tgf-β-induced Col1a1, Mmp2 and α-Sma expression in human LX-2 cells. These data suggested that DA treatment reduced liver damage through development of a hepatic adaptive response and inhibition of the activation of HSCs, which led to a reduction in liver fibrosis formation in BDL mice.

**Conclusions:**

DA treatment protected against liver damage and fibrosis following BDL and might be an effective therapy for extrahepatic cholestasis due to bile duct obstruction.

## INTRODUCTION

Obstructive cholestasis is caused by occlusion of the common bile duct or its tributaries and is associated with reduced bile flow, hepatic accumulation of bile acids, progressive liver injury and development of fibrosis. Production and accumulation of extracellular matrix (ECM) constituents is the primary cause of liver fibrosis [1]. Furthermore, activation of hepatic stellate cells (HSCs) can result in increased ECM synthesis through the actions of hepatic fibrolytic matrix metalloproteinases and convert liver parenchyma into scar tissue, leading to liver cirrhosis [2]. However, aside from liver transplantation, effective medical treatments for cirrhosis are not currently available. Therefore, effective inhibition of HSC activation is a promising therapeutic target to protect the liver against fibrosis in cholestatic liver diseases.

To date, ursodeoxycholic acid (UDCA) is the only FDA-approved drug to treat cholestatic liver disorder in the clinic. However, its effectiveness is limited for primary biliary cirrhosis (PBC) patients [3]. Although PBC has received attention in recent years to study the molecular mechanism of cholestasis, new therapeutic approaches have been not developed. Thus, there is a pressing need to develop a safe and effective agent that may facilitate successful treatments for cholestatic patients.

For centuries, medicinal plants have traditionally been used to treat liver diseases, and the toxicity factor appears to be low, with few side effects [4–7]. AP, also known as Chuan-xin-lian in Chinese, is a Chinese medicinal herb widely used to treat infection, inflammation, the common cold, fever, diarrhea, hypertension, cardiovascular and liver disorders [8–11]. In the last several years, increasing attention has been paid to AP because of its diverse therapeutic properties. The reported primary active ingredients of AP are several diterpene lactones, flavonoids, and polyphenols [12, 13]. Andrographolide and dehydroandrographolide are diterpenoid compounds that are believed to be the main contributors to the therapeutic properties of AP. Andrographolide has shown protective effects against liver damage caused by carbon tetrachloride [14], acetaminophen [15] and hexachlorocyclohexane [16]. DA (14-deoxy-11, 12-didehydroandrographolide, C_20_H_28_O_4_) has also been shown to play a role in the plant's pharmacological effects, specifically exhibiting anti-inflammatory, anti-cancer, anti-platelet and anti-hypertension activities in various cells and animals [11, 17–19]. Recently, a study reported that DA possessed free radical scavenging and ferric reducing capacities [9, 20]. However, the hepatoprotective effects of DA remain unclear. Given that AP and andrographolide have hepatic protective effect in human and animal model, DA may be an attractive candidate drug for the treatment of cholestatic liver diseases.

In this study, we tested the hepatic protective effects of DA *in vivo* in a mouse model of obstructive cholestasis and *in vitro* in human LX-2 cells. Our findings demonstrate that DA treatment significantly reduced liver damage and fibrosis formation following BDL in mice. In further elucidating the underlying molecular mechanisms, we found that the effectiveness of DA treatment is due to its ability to enhance the hepatic adaptive response, inhibit HSC activation and decrease ECM deposition, which will likely lead to reduced liver fibrosis formation in cholestatic disorders.

## RESULTS

### DA treatment reduced BDL-induced liver injury in mice

**Figure 1:**
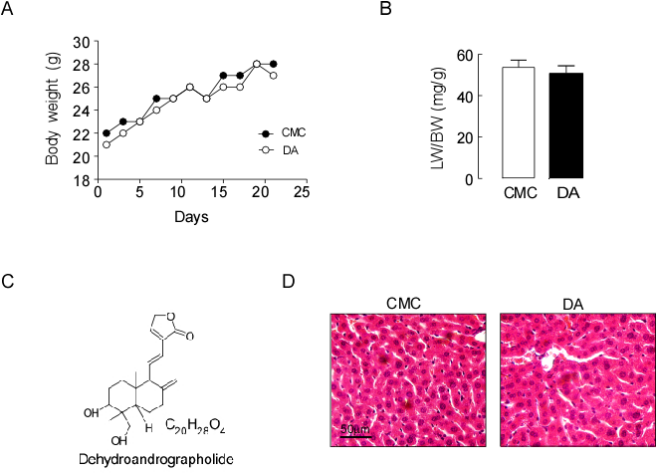
DA treatment had no effect on the liver of mice **(A)** Body weights of mice receiving either CMC or DA treatment. **(B)** The ratios of liver/body weight and **(C)** The chemical structure of DA. **(D)** H&E-stained liver histology. Data represent the means ± SEM of 3 independent experiments. n=10, *p*>0.05.

**Figure 2:**
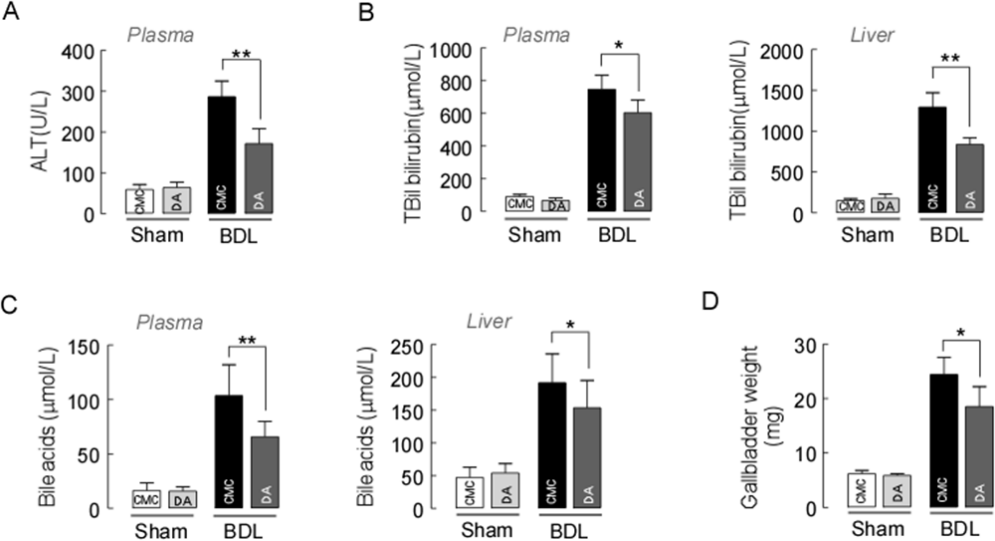
Effect of DA on BDL-induced liver injury **(A)** Plasma ALT, **(B)** total bilirubin and **(C)** total bile acid levels were significantly reduced in DA-BDL mice compared to CMC-BDL mice. **(D)** DA-treated mice also exhibited significantly smaller gallbladder weights than CMC-treated BDL mice. Data represent the means ± SEM of 3 independent experiments. n=10, ^*^ *p*<0.05 and ^**^p<0.01, between the indicated groups.

Figure 1A depicts the body weights and the ratios of liver/body weight (Figure 1B) of mice receiving either CMC or DA (the chemical structure is shown in Figure 1C) at a high dose of 100 mg/kg body weight. This dose was considered one tenth of the proposed oral LD_50_ mg/kg body weight obtained from the acute toxicity study. No changes in sizes, gross anatomical features or weights were observed (*p*>0.05). H&E staining demonstrated that the livers of both groups of mice exhibited similar histological features, as well as comparable liver lobule structures and hepatocyte densities (Figure 1D). This suggested that DA treatment had little toxicity in normal mice. We observed the levels of markers associated with hepatobiliary disease, including ALT, TBIL, TBA, TC and LDL-C in serum and/or liver tissues in sham and BDL mice. Biochemical analysis revealed that DA dose at 100 mg/kg body weight did not change the serum TBIL, TBA, ALT, TC or LDL-C levels in sham mice, but there were significantly reduced serum levels of ALT, TC and LDL-C in BDL mice compared to CMC-BDL mice (Figure 2A and Supplementary Figure 1, *p*<0.01 and *p*<0.05). Furthermore, DA treatment at 100 mg/kg body weight decreased TBIL (Figure 2B, *p*<0.05 or *p*<0.01) and TBA levels (Figure 2C, *p*<0.05 or *p*<0.01) in serum and liver tissues in BDL mice. In addition, the weight of the gallbladder was also observed in BDL mice. As shown in Figure 2D, mice treated with DA at 100 mg/kg body weight exhibited significantly lower gallbladder weights than mice treated with CMC, suggesting that bile production was decreased in DA-BDL mice. These findings indicate that DA treatment improved the overall condition of hepatobiliary system of the cholestatic mice.

### DA treatment improved liver histology in BDL mice

**Figure 3:**
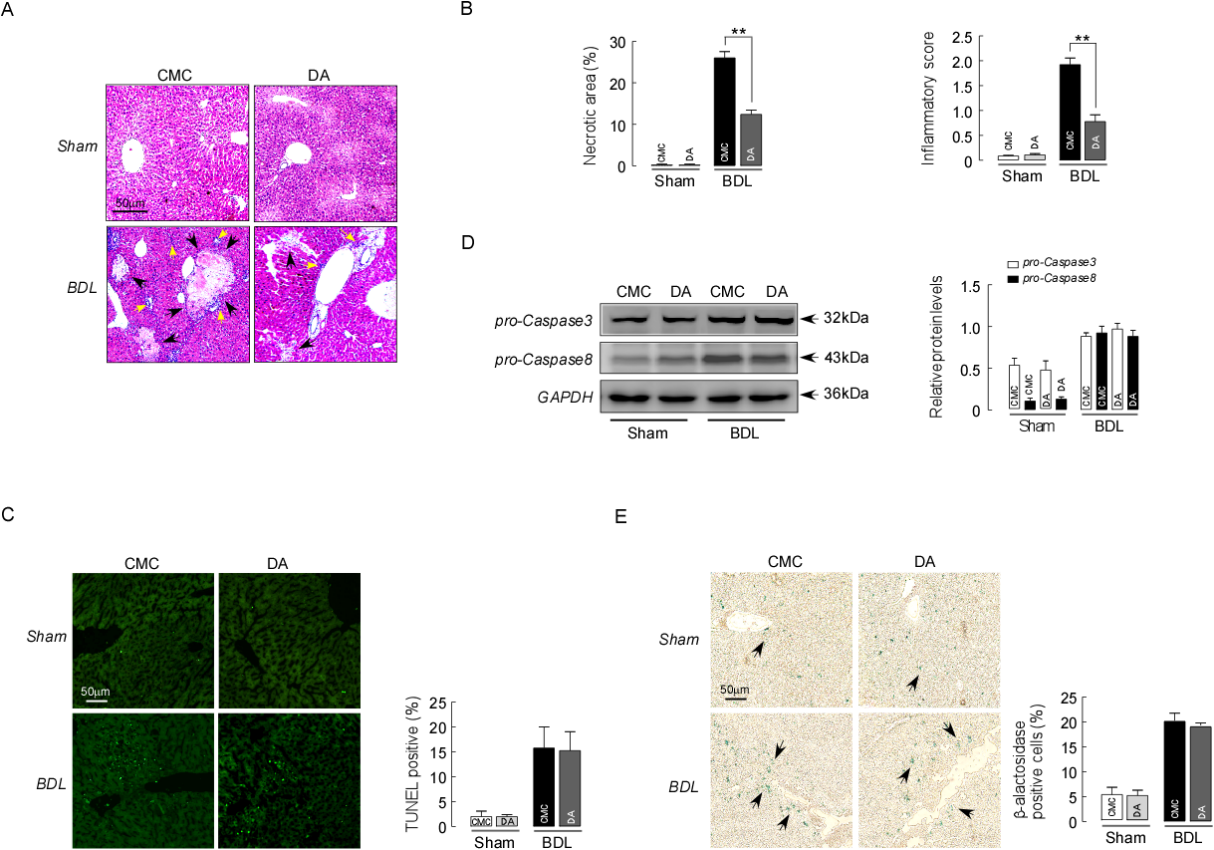
DA improved liver histology in BDL mice **(A)** Representative images of H&E-stained liver sections from sham and BDL mice treated with either CMC or DA. Liver necrosis is shown by black arrows. Yellow arrows point toward inflammatory cell. **(B)** Scores of the double-blind assessment of liver necrotic areas (left) and inflammatory cell infiltration (right) of sham and BDL mice treated with CMC or DA. n=10, ^**^ *p*<0.01, between the indicated groups. **(C)** Representative images of TUNEL-stained liver sections from sham and BDL mice treated with either CMC or DA (left). TUNEL-positive cell counts in the bile necrotic zones of sham and BDL mice treated with CMC or DA (right). Data represent the means ± SEM of 3 independent experiments. n=10, *p*>0.05. **(D)** Representative blots and densitometry data of procaspase3 and procaspase8 expression in BDL mice livers with and without DA treatment. Data represent the means ± SEM of 3 independent experiments. n=3, *p*>0.05. **(E)** Representative images of β-alactosidase-stained liver sections from sham and BDL mice treated with either CMC or DA. Senescence cell is shown by arrows. Data represent the means ± SEM of 3 independent experiments. n=10, *p*>0.05.

DA treatment at 100 mg/kg body weight in BDL mice reduced hepatocellular ballooning, the foci of liver bile necrosis in the periportal regions and lobular infiltration by neutrophils, determined by H&E staining (Figure 3A). Furthermore, DA at 100 mg/kg significantly decreased the area of bile necrosis in the parenchyma in BDL mice compared to CMC-BDL mice. As quantified by morphometric analysis of bile necrotic areas, we also observed significantly decreased liver damage in DA-BDL mice compared to CMC-BDL mice (Figure 3B, *p*<0.01). To accurately clarify whether biliary necrosis were caused by hepatocyte apoptosis or necrosis, TUNEL staining was performed for BDL mice. Figure 3C shows that TUNEL-positive cells were not increased in the bile necrotic zones in CMC-BDL mice compared with DA-BDL mice (*p*>0.05). Immunoblot analysis showed that expression of Caspase 3 and Caspase 8 were not changed by DA treatment in BDL mice compared to CMC-BDL mice (Figure 3D, *p*>0.05). In addition, we also examined the effect of DA on cellular senescence using β-galactosidase staining in BDL mice liver. The number of β-alactosidase positive cells was significantly increased in BDL mice than in sham mice. But no different was seen in β-alactosidase positive cells number between DA-BDL mice and CMC-BDL mice (Figure 3E, *p*>0.05), suggesting that hepatocyte death following BDL was primarily induced by cell necrosis. However, DA at 50 mg/kg body weight nether altered serum levels of ALT and TBIL nor reduced biliary necrotic areas and periportal inflammation in BDL mice liver. Thus we chose a DA dose at 100 mg/kg for our studies.

### DA enhanced the adaptive response to BDL in mice

**Figure 4:**
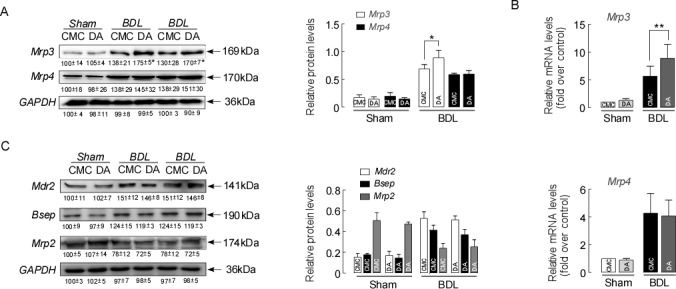
DA enhanced the adaptive response to BDL in mice **(A)** Representative blots and densitometry data of Mrp3 and Mrp4 expression in BDL mouse livers with and without DA treatment. n=5, ^*^p<0.05 BDL-CMC versus BDL-DA. **(B)** Relative mRNA expression for Mrp3 and Mrp4 in BDL mice. n=3, ^**^p<0.01, between indicated groups. **(C)** Representative blots and densitometry data of Mdr2, Bsep and Mrp2 expressions in BDL mice livers with and without DA treatment. Data are expressed in arbitrary units. n=3. Data represent the means ± SEM of 3 independent experiments.

The extent of liver damage in obstructive cholestasis is regulated by adaptive changes in hepatic membrane transporters [24, 25]. We assessed whether DA treatment altered the expression of hepatic efflux transporters in BDL mice. Figure 4A demonstrates that the expression of Mrp3 and Mrp4 (mainly basolateral transporters) was up-regulated in CMC-BDL mice compared to sham mice, according to a western blot assay. However, Mrp3 was further up-regulated in DA-BDL mice with the highest elevation at a DA dose of 100 mg (*p*<0.05). Examination of the gene expression of Mrp3 and Mrp4 also confirmed that DA further increased the levels of Mrp3 mRNA compared to the levels in CMC-BDL mice (Figure 4B, *p*<0.01). Assessment of the expression of canalicular efflux transporter proteins revealed increased Mdr2 and Bsep expression, whereas decreased Mrp2 protein expression was seen in all BDL mice. However, there was no statistically significant difference after DA treatment (Figure 4C, *p*>0.05). These results suggested that up-regulation of Mrp3 exerted an effective adaptive response to BDL-induced liver injury as evidenced by the reduction of bile infarcts in DA-BDL mice.

### DA treatment inhibited liver fibrosis formation in BDL mice

**Figure 5:**
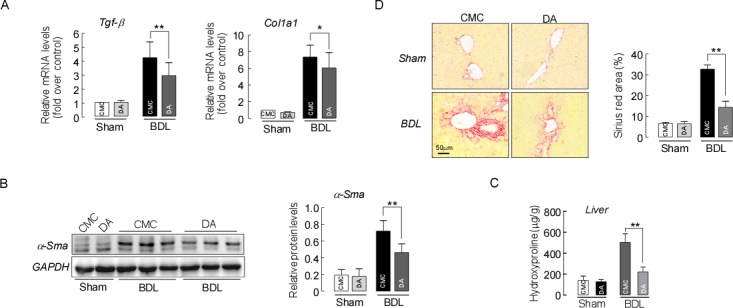
DA treatment inhibited liver fibrosis formation in BDL mice **(A)** Relative liver mRNA expression for Tgf-β, α-Sma and Col1a1. Data are expressed as the fold change relative to CMC-mice. Data were normalized to β-actin. **(B)** Liver α-Sma protein expression detected by western blotting. Data were normalized to GAPDH. **(C)** Liver hydroxyproline levels. **(D)** Representative images of sirius red staining of liver sections from sham and BDL mice treated with either CMC or DA. Data represent means ± SEM of 4 independent experiments. n=3-5, ^*^ *p*<0.05 and ^**^p<0.01, between indicated groups.

The BDL model in mice leads to liver fibrosis via activation of the expression of hepatic fibrogenesis genes [26]. To further determine if this mechanism contributed to the improvement in liver injury in DA-BDL mice, we next evaluated whether DA treatment could affect hepatic expression of profibrogenic genes. qPCR demonstrated that the expression of the key genes Tgf-β, α-Sma and Col1a1 in hepatic fibrogenesis were significantly decreased in DA-BDL mice compared to CMC-BDL mice (Figure 5A, *p*<0.05 or *p*<0.01). A western blot analysis also revealed a significant reduction in α-Sma protein expression in DA-BDL mice compared to CMC-BDL mice (Figure 5B, *p*<0.01). Furthermore, hydroxyproline analysis showed significantly lower levels of this fibrosis marker in the livers of DA-BDL mice compared to the levels in livers of CMC-BDL mice (Figure 5C, *p*<0.01). This result was also supported by analysis of Sirius Red staining of the liver, which revealed reduced collagen deposits in the periportal area five days after BDL in DA-BDL mice compared with CMC-BDL mice (Figure 5D, *p*<0.01). These data suggest that DA treatment represses the activation of HSCs, further reducing fibrosis formation in BDL mice.

**Figure 6:**
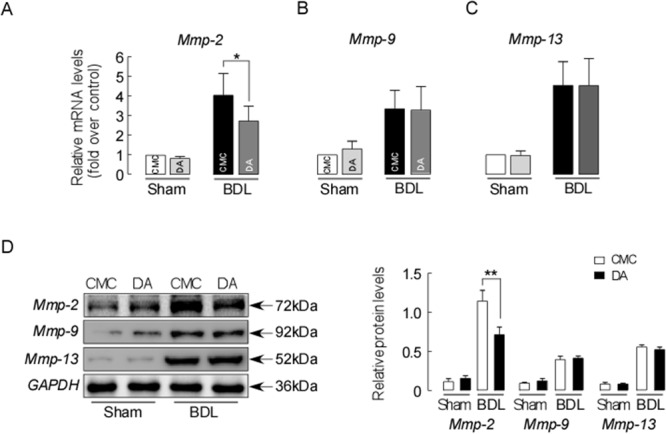
DA treatment decreased ECM deposition in BDL mice **(A)** Relative Liver mRNA expression for Mmp-2, Mmp-9 **(B)** and Mmp-13 **(C)** in sham and BDL mice. Data were normalized to β-actin. **(D)** Liver Mmp-2, Mmp-9 and Mmp-13 protein expressions detected by western blotting. Data were normalized to GAPDH. Data are expressed in arbitrary units. Data represent the means ± SEM of 3 independent experiments. n=3, ^*^ *p*<0.05, ^**^ *p*<0.01, between the indicated groups.

The anti-fibrotic effect of DA was further verified by the action of hepatic fibrolytic matrix metalloproteinases. As shown in Figure 6, the mRNA expressions of Mmp-2 (A), Mmp-9 (B) and Mmp-13 (C) were significantly up-regulated in BDL mice compared to sham mice. However, DA treatment in BDL mice did not alter the mRNA level of Mmp-9 and Mmp-13, whereas the mRNA level of Mmp-2 was significantly decreased compared to that of CMC-BDL mice (*p*<0.01). Consistent with the mRNA levels, Mmp-2 protein expression level was greatly reduced in DA-BDL mice than CMC-BDL mice (Figure 6D, *p*<0.01). These data indicate that the liver protective effect exerted by DA treatment might be meditated through reduced Mmp secretion, decreasing ECM deposition following BDL.

### DA treatment reduced col1a1 and mmp2 expression in LX-2 cells

**Figure 7:**
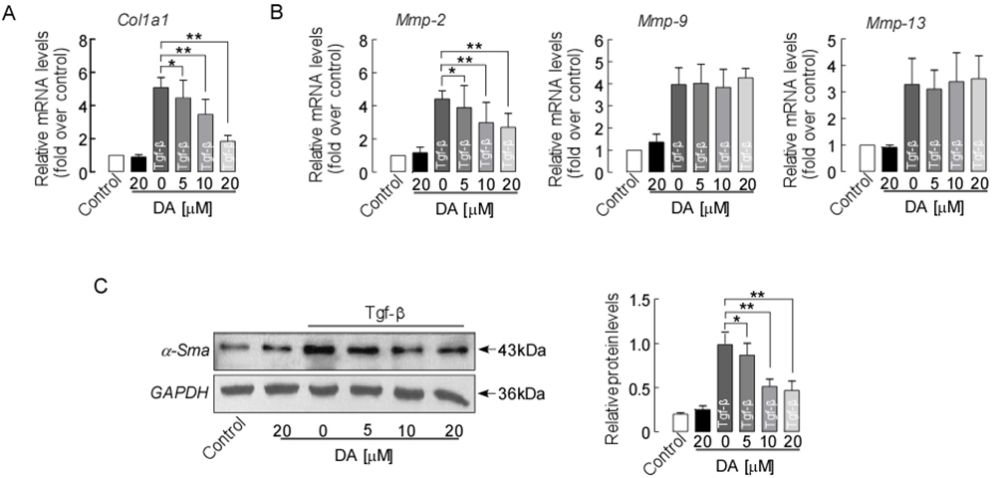
DA treatment reduced the expression of fibrotic marker genes/protein in human LX-2 cells **(A)** mRNA expression of Col1a1, **(B)** Mmp2, Mmp-9, and Mmp-13 in human LX-2 cells. **(C)** α-Sma protein expression detected by western blotting. Data are normalized to GAPDH. Data represent means ± SEM of 4 independent experiments. n=3, ^*^ *p*<0.05 and ^**^ *p*<0.01 between indicated groups.

Because DA treatment reduced markers of hepatic fibrosis in BDL mice, we next assessed if this treatment would have the same anti-fibrotic effects in human LX-2 cells. LX-2 cells were incubated with DA at 5, 10, and 20 μM (IC_50_ 10 μM) and Tgf-β for 24 h [27–29]. Figure 7A shows that 24 h of treatment with 10 and 20 μM DA significantly inhibited Tgf-β-induced Col1a1 mRNA expression (*p*<0.01). DA treatment with 10 and 20 μM also further reduced Tgf-β-stimulated Mmp2 mRNA expressions in LX-2 cells (Figure 7B, *p*<0.01). However, Mmp9 and Mmp13 were not changed by DA treatments at any dose. Western blot analysis also showed significant reduction in ɑ-Sma protein expression in cells after 10 and 20 μM DA treatment (Figure 7C, *p*<0.01). Moreover, low dose DA at 5 μM altered the Tgf-β-induced expression of Col1a1, Mmp2 and ɑ-Sma expression (*p*<0.05) in LX-2 cells, suggesting that DA exerts its anti-fibrotic effects in the liver through inhibition of Col1a1, ɑ-Sma, and Mmp2 expression.

## DISCUSSION

In this study, we assessed the liver protective effects of DA *in vivo* in a BDL cholestatic mouse model and *in vitro* in human LX-2 cells. DA treatment attenuated liver damage in the BDL model as demonstrated by 1) improved mouse liver function and histology and reduced liver bile infarction, necrosis and fibrosis (Figure 2&3); 2) enhanced liver adaptive response to BDL by up-regulating Mrp3 expression (Figure 4); and 3) reduced liver hydroxyproline content and fibrotic markers (Figure 5&6). Moreover, the reduced liver fibrogenesis upon DA treatment was further confirmed in human LX-2 cells (Figure 7).

BDL-induced cholestasis results in the loss of bile excretory function and toxic bile accumulation, leading to injury of the liver parenchyma [30]. However, adaptive response mechanisms can compensate for the extent of liver injury resulting from cholestasis [31, 32]. In cholestasis, to compensate, the main influx transporters of the basolateral membrane of hepatocytes, including sodium/bile acid and sulfated solute cotransporter1 (Slc10a1) and organic anion transporter (Slco1a1), are down-regulated, whereas main efflux transporters of the basolateral membrane of hepatocytes, including Mrp3, Mrp4 and Ostα/β, are up-regulated to reduce or accelerate the uptake or secretion of bile acids and other organic anions [33]. In addition, down-regulation of the canalicular membrane transporters of hepatocytes, Bsep and Mrp2, is associated with cholestasis [24, 34]. In the present study, DA treatments were able to efficiently relieve BDL-induced liver injury by modifying hepatocellular gene and protein expression of Mrp3 compared to CMC-BDL mice (Figure 4A&4B). Up-regulation of Mrp3 can decrease bile acid accumulation, resulting in hepatocyte damage. This result was supported by our experimental results examining serum enzyme markers and liver histology (Figure 2). Although ductular reaction may represent an evacuation route for bile during cholestasis [35], our data did not show that DA treatments significantly enhanced bile duct proliferation, as assessed by Ck19 staining (data not shown). Thus, it is likely that DA treatments exert a cytoprotective effect in the liver directly through enhancing liver adaptive mechanisms to BDL, e.g., up-regulation of Mrp3 expression.

AP, a Chinese medicinal herb, has been commonly used for liver disorder therapy in China. Andrographolide, one of the main ingredients in AP, has been shown to have a liver-protective effect in various animal models [14–16]. DA is another active ingredient of AP. However, studies on the effects of DA in patients and animal with liver disorder are limited. In a recent study, DA was shown to have anti-oxidant properties [20], which may render protective effects against oxidative liver damage and fibrosis. To extend this observation in liver disorders, we tested the efficacy of DA in inhibiting the progression of cholestatic liver fibrosis induced by BDL in mice. Data from our study demonstrate a significant decrease in collagen deposition by DA treatments in the livers of BDL mice (Figure 6). Moreover, this result was further supported by biochemical and gene expression analyses, as markers such as hydroxyproline content, expression of Tgf-β, α-Sma and Col1a1 in the livers of DA-treated mice were significantly decreased compared to those in CMC-treated BDL mouse livers (Figure 5), suggesting that DA attenuated liver fibrosis and that this effect was companied by suppressed activation of HSCs. Moreover, the anti-fibrotic effect of DA was also evidenced by reduced expression of Mmp2 in the BDL model (Figure 6). Although DA has never been tested in clinical patients, we used a human hepatic stellate cell line to determine whether it would show the same response to DA. We treated human LX-2 cells with DA at different concentrations. Low dose (5 μM) DA had beneficial effects and altered the Tgf-β-induced expression of Col1a1, Mmp2 and α-Sma (Figure 7), and these findings were consistent with the results in the animal studies. Tgf-β targets HSCs of the liver and helps to induce the transdifferentiation of HSCs into fibrogenic myofibroblasts, promoting the formation of liver fibrosis [36–39]. Thus, the suppression of liver fibrosis progression by DA observed in human LX-2 cells was likely mediated by preventing activation of Tgf-β profibrogenic pathways.

In conclusion, our results reveal that DA reduced liver damage and fibrosis and promoted the development of a hepatic adaptive response in a mouse model of cholestasis and in human LX-2 cells *in vitro*, suggesting that DA, a natural compound, is an attractive candidate to treat patients with chronic cholestatic disorders.

## MATERIALS AND METHODS

### Materials

DA was purchased from the National Institution for the Control of Pharmaceutical and Biological Products in China, and its purity (> 99%) was defined by HPLC. All chemical reagents were obtained from Sigma-Aldrich (St Louis, MO, USA). ALT, low density lipoprotein cholesterol (LDL-C) and total cholesterol (TC) analysis kits were purchased from Nanjing Jiancheng Bioengineering Institute (Nanjing, China). Total bilirubin (TBIL) and total bile acid (TBA) ELISA kits were purchased from Huamei Bioengineering Institute (Wuhan, China). Polyclonal antibodies against Mrp2, Mrp3, Mrp4 and Bsep antibody were obtained from Santa Cruz Biotechnologies (Santa Cruz, CA, USA). α-Sma, Mmp2, Mmp9, Mmp13 and GAPDH antibodies were obtained from Abcam (Cambridge, UK). TRIzol reagent, primer sequences for a TaqMan real-time PCR kit (Mmp2, Mmp9 and Mmp13) and secondary antibodies were purchased from Invitrogen (Carlsbad, CA, USA). Tgf-β was purchased from R&D (Minneapolis, MN, USA). Quantitative polymerase chain reaction (qPCR) kit was obtained from Roche (Indianapolis, IN, USA). ECL was obtained from Thermo Scientific (Rockford, IL, USA).

### Animals and experimental protocols

Adult male C57BL/6J mice (8-10 weeks old) were used for this study. The mice were maintained in a specific pathogen-free grade animal facility under a 12-h light-dark cycle. All procedures were approved by the Committee on Animal Research of Jinzhou Medical University and followed the ARRIVE guidelines pertaining to animal experimentation [21]. Mice were randomized and pretreated with DA by gavage at a dose of 100 mg/kg body weight or vehicle for 5 days (drug concentration equilibrium) prior to bile duct ligation, and then DA treatment continued for 7 days. The vehicle-treated control mice received an equal volume of 0.5% CMC (10 ml/kg). Total duration for DA treatment was 12 days. BDL was performed via double ligation and section of the common bile duct in mice. Briefly, mice were fasted (not water) overnight prior to the operation. Mice were anesthetized with 2% isoflurane inhalation and were fixed on an animal operating table with a heating pad. After midline laparotomy, the common bile duct was isolated from the surrounding tissue and dissected between two ligature knots with 8-0 nylon suture. Sham mice underwent the same procedure, including the mobilization of the bile duct, but without dissection and ligation. Mice were placed on a heating pad while recovering from the anesthesia [22]. Serum and liver tissue were collected from the BDL and sham mice and stored at −80°C.

### Liver function analysis

Plasma ALT, TC and LDL-C were measured as an indicator of hepatic injury using a commercial kit. TBA concentration and TBIL levels in the plasma and liver were determined using commercial ELISA kits.

### Histopathology and immunostaining

Mice were anesthetized using an over-dose of phenobarbital sodium via intraperitoneal injection. Then, the livers were removed and fixed in 4% paraformaldehyde for 24 h at 20°C. The samples were dehydrated using increasing concentrations of ethanol and embedded in paraffin. The sections were deparaffinized and stained using hematoxylin and eosin (H&E) and Sirius Red. Liver histology was blindly assessed for necrosis and fibrosis on a 1- 4+ scale.

### Cells culture and treatment

The human LX-2 cell line was purchased from the Shanghai Institute of Biological Science (Shanghai, China). The cells were grown in plastic culture flasks under standard conditions (37°C with 5% CO_2_ in a completely humidified atmosphere) using DMEM supplemented with 10% heat-inactivated FBS and 1% penicillin/streptomycin. Cell detachment was achieved by rinsing with 0.05% trypsin/0.02% EDTA solution. After 24 h of attachment, the cells were cultured in serum-free DMEM for 24 h and then treated with DA at an IC_50_ concentration and two difference concentrations or with Tgf-β at 2 ng/ml for 24 h to assay the hepatic fibrogenesis genes. DA was dissolved in dimethyl sulfoxide (DMSO) to form a 20 mM stock solution, which was stored at −20°C and diluted to the desired final concentration in DMEM at the time of use.

### Quantitative real-time PCR and western blot analysis

Total RNA was extracted from liver samples via homogenization in TRIzol reagent. cDNA was synthesized from 2 μg of total RNA using random hexamer primers and 200 U of Moloney murine leukemia virus reverse transcriptase for 1 h at 37°C. qPCR was performed using a LightCycler with a FastStart DNA MasterPLUS SYBR Green I kit. β-Actin was used as a reference gene. The results are reported as normalized and calibrated ratios. The primers were as follows: 5‘-CCACCATGTACCCAGGCATT-3′ (sense) and 5′-CCGGACTCATCGTACTCCTG-3′ (antisense) for β-actin; 5′-GAAACCCGAGGTATGCTTGA-3′ (sense) and 5′-GACCAGGAGGACCAGGAAGT-3′ (antisense) for Col1a1; 5′-GTCAGACATTCGGGAAGCAG-3′ (sense) and 5′-GCGTATCAGTGGGGGTCA-3′ (antisense) for Tgf-β. Mmp2, Mmp9 and Mmp13 were examined using an ABI kit.

Liver total proteins and membrane proteins were isolated from the mouse liver tissues. Western blot analysis was performed as previously described with some modifications [23]. Briefly, samples were loaded onto an 8-10% SDS-polyacrylamide gel (20 μg of protein/lane) for electrophoresis, and proteins were transferred to a polyvinylidene difluoride membrane. The membrane was subsequently incubated with a primary antibody overnight at 4°C. Following washing with TBS, the membrane was incubated with a horseradish peroxidase (HRP)-linked secondary antibody at room temperature for 1 h in the dark. The bands were developed using enhanced chemiluminescence. The relative quantities of protein expression were analyzed using Image J software.

### Statistical analysis

The data are shown as the mean ± standard error of the mean (SEM). All experiments were performed in triplicate. All data were estimated using Student's t-test or chi square test for two-group data sets and one-way ANOVA followed by Bonferroni's post hoc test to compare more than two groups, using GraphPad Prism software, version 5.0. A p value < 0.05 was considered to be statistically significant.

## SUPPLEMENTARY MATERIALS FIGURE


