# Supplementary material for: Protective effect of dehydroandrographolide on obstructive cholestasis in bile duct-ligated mice
Source: Oncotarget. 2017 Sep 23;8(50):87903–13. doi: 10.18632/oncotarget.21233 (PMC5675681; doi:10.18632/oncotarget.21233)
Supplement: Supplementary file 1 [file oncotarget-08-87903-s001.pdf]

Protective effect of dehydroandrographolide on obstructive cholestasis in bile duct-ligated mice

SUPPLEMENTARY MATERIALS

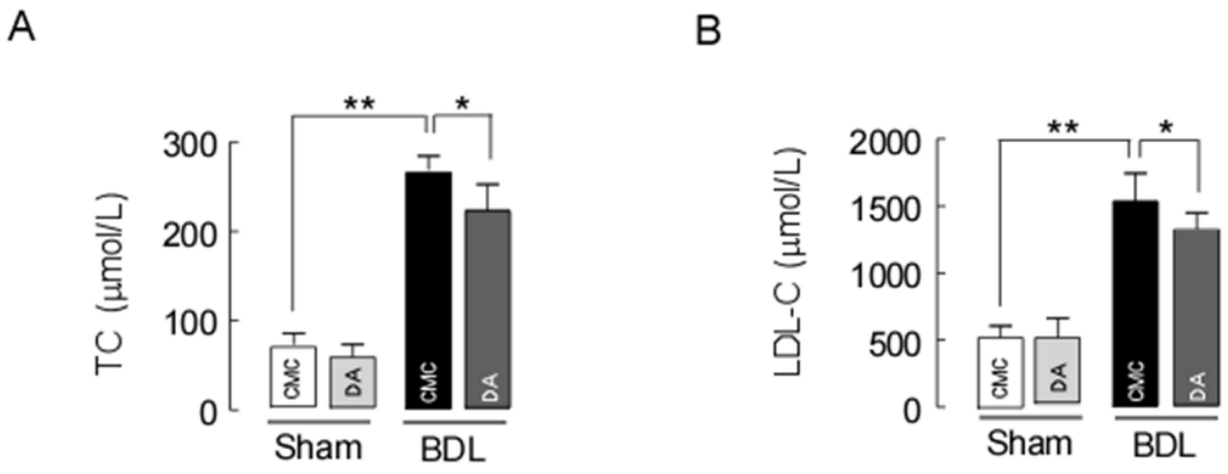

Supplementary Figure 1: DA treatment reduced BDL-induced liver injury. **(A)** Plasma TC, **(B)** LDL-C levels were significantly reduced in DA-BDL mice compared to CMC-BDL mice. Data represent means ± SEM of 3 independent experiments. n=10, *$p<0.05$ and **$p<0.01$ between indicated groups.
